# Organofluorine Mass Balance Analysis of Whole Blood Samples in Relation to Gender and Age

**DOI:** 10.1021/acs.est.1c04031

**Published:** 2021-09-14

**Authors:** Rudolf Aro, Ulrika Eriksson, Anna Kärrman, Leo W. Y. Yeung

**Affiliations:** Man-Technology-Environment (MTM) Research Centre, School of Science and Technology, Örebro University, Örebro SE-701 82 Sweden

**Keywords:** organofluorine mass balance analysis, per- and polyfluoroalkyl substances, blood unidentified organofluorine, extractable organofluorine

## Abstract

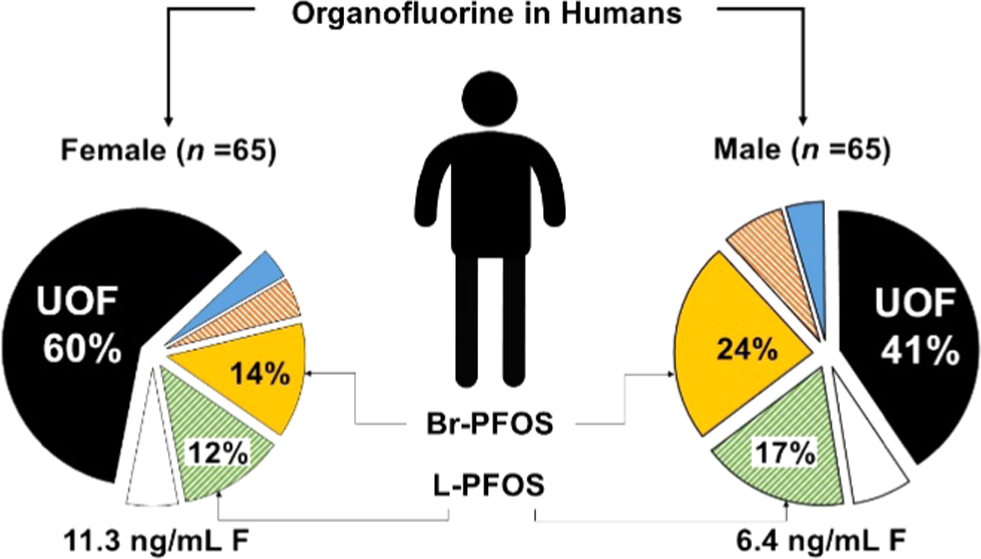

Studies have highlighted the increasing fraction of unidentified organofluorine (UOF) compounds in human blood, whose health effects are not known. In this study, 130 whole blood samples from the Swedish general population were analyzed for extractable organofluorine (EOF) and selected per- and polyfluoroalkyl substances (PFAS). Organofluorine mass balance analysis revealed that 60% (0–99%) of the EOF in female samples could not be explained by the 63 monitored PFAS; in males, 41% (0–93%) of the EOF was of unidentified origin. Significant differences between both age groups and gender were seen, with the highest fraction of UOF in young females (70% UOF, aged 18–44), which is contrary to what has been reported in the literature for commonly monitored compounds (e.g., perfluorooctane sulfonic acid, PFOS). Increasing the number of monitored PFAS did not lead to a large decrease of the UOF fraction; the seven highest PFAS (C8–C11 PFCAs, C6–C8 PFSAs) accounted for 98% of sum 63 PFAS. The high fraction of UOF in human samples is of concern, as the chemical species of these organofluorine compounds remain unknown and thus their potential health risks cannot be assessed.

## Abstract 2

Organofluorine mass balance analysis of blood revealed a large fraction of unidentified organofluorines, which was especially pronounced for young females.

## 1 Introduction

The most studied per- and polyfluoroalkyl substances (PFAS), perfluorooctanoic acid (PFOA) and perfluorooctane sulfonic acid (PFOS), have been linked to various negative health outcomes. In the general human population, PFOA has been associated with changes in birth weight^1,2^ and immunotoxicity.^3^ In occupationally exposed, PFOA has been linked to prostate cancer, cerebrovascular disease, diabetes,^4^ and ulcerative colitis.^5^ PFOS has been linked to endocrine disruption,^6,7^ reduced sperm quality,^8^ and clinical chemistry effects.^9,10^

One of the main producers, 3M phased out PFOS and PFOA production in the United States after 2002,^11^ and both PFOS and PFOA have been included in the Stockholm convention since 2009 and 2019, respectively.^12^ As a result, production has shifted to other PFAS, for example, shorter (C4 and C6)-chained PFAS^13^ and perfluoroether compounds.^14^ While PFAS with shorter perfluorinated carbon backbones were considered to have a lower health and environmental impact due to lower bioaccumulative potential,^15^ recent studies have indicated that at higher concentrations the PFAS with shorter carbon backbones have toxicities similar to their longer-chained analogues.^16,17^ Human biomonitoring studies have already detected other ether compounds (6:2 chlorinated polyfluorinated ether sulfonate (6:2 Cl-PFESA) and 4,8-dioxa-3H-perfluorononanoic acid (ADONA)).^18,19^

In 2018, OECD identified more than 4700 PFAS-related CAS numbers,^20^ and in 2021, OECD has defined PFAS as fluorinated substances with at least a single fully fluorinated methyl or methylene carbon atom (without any H/Cl/Br/I atom attached to it).^21^ The number of PFAS present in a sample is likely higher due to the degradation intermediates of perfluoroalkyl acid (PFAA) precursors;^22^ the degradation pathways are not known for all PFAS. At the same time, most human biomonitoring studies are looking at only around a dozen PFAS,^23^ which is several orders of magnitude less than the number of PFAS humans could potentially be exposed to. Several tools are available to address this knowledge gap, for example, nontarget screening (NTA),^24^ total oxidizable precursor (TOP) assay,^25^ and organofluorine mass balance analysis.^26^ Each of these has unique advantages and drawbacks. NTA relies on data mining tools and yields only semiquantitative results.^27^ TOP assay assumes that the unknown compounds will oxidize to PFAAs, which is not always the case.^28^ Organofluorine (OF) mass balance analysis, in comparison, only requires the measurement of OF that can be extracted from a sample (extractable organofluorine (EOF)) and separated from inorganic fluorine (IF), in addition to target PFAS analysis.

The amount of EOF can be compared to the amount of fluorine attributable to the target PFAS in the sample; the difference is assumed to originate from unidentified organofluorine (UOF) compounds. Since only a handful of OF compounds have been found in nature,^29^ the UOF is assumed to be of anthropogenic origin. All PFAS contain fluorine, and a high concentration of a yet to be identified PFAS (not included in target analysis) would result in an increased EOF concentration. However, the EOF could also contain organofluorine compounds other than PFAS, for example, fluorinated pharmaceuticals.^30^ Organofluorine mass balance analysis has been used to show the widespread presence of UOF in various matrices, from water to invertebrates.^26,31^ Previous studies into the organofluorine mass balance of human samples have indicated the presence of UOF in humans as well.^32,33^ More worryingly, the UOF fraction in human samples has been increasing,^34^ simultaneously with a decrease in the levels of target PFAS (e.g., PFOA and PFOS).^35,36^

A common method for EOF determination, necessary for OF mass balance analysis, is combustion ion chromatography (CIC). In CIC, the sample extract is combusted at a high temperature and the formed fluoride is measured to determine the fluorine content of the sample. Since the whole sample extract is combusted, it cannot discriminate between different PFAS classes as long as they are extracted during sample preparation. The EOF analysis will include both unknown PFAS and those compounds that are otherwise difficult to measure using mass spectrometric techniques. This robustness comes at the cost of losing any structural information regarding the compounds in the sample. The method also lacks the capability to differentiate between IF and OF; they have to be separated during sample preparation. Different studies have made use of various extraction methods, for example, protein precipitation with acetonitrile^37^ and ion-pair extraction (IPE).^32^ This complicates comparison between studies, as each different extraction method will result in a different fraction of EOF. An additional challenge to EOF analysis is the high limit of detection (LOQ) of the CIC analysis, requiring higher sample amounts compared to target PFAS analysis.

The aim of this study was to assess the level of UOF exposure in the general population in Sweden and identify which PFAS are driving the EOF exposure, ultimately providing guidance for biomonitoring studies. The influence of gender and age on the proportion of UOF was also investigated. EOF and PFAS were measured in 148 whole blood samples from Sweden. A total of 63 PFAS were analyzed for the OF mass balance (PFAAs, their precursors, and some known PFOS and PFOA replacement compounds).

## 2 Materials and Methods

Details on chemicals are given in the Supporting Information.

### 2.1 Analytical Standards

Most native and all isotope-labeled internal standards (IS) were purchased from Wellington Laboratories (Guelph, Canada). The exceptions were 10:2 fluorotelomer phosphate mono- and di-esters (10:2 monoPAP and 10:2 diPAP, purchased from Chiron (Trondheim, Norway)), trifluoroacetic acid (TFA, purchased from Merck KGaA (Darmstadt, Germany)), and perfluoropropanoic acid (PFPrA, purchased from Sigma-Aldrich). Details on the chemicals used in this study are given in SI 1, a list of native standards is given in SI 4 Table S1, and internal standards are listed in SI 5 Table S2.

### 2.2 Sample Collection

A total of 148 whole blood samples were collected between 2018 and 2019 from people donating blood; all participants gave written informed consent; this work was approved by the Ethics Committee in Uppsala (decision: DNR 2018/158). The samples (30 at each location) were collected from Umeå, Uppsala, Stockholm, Örebro, and Malmö to achieve a wide geographic coverage (additional information in SI 2 Figure S1). Each participant gave 4–9 mL of whole blood, collected in EDTA or heparin vacutainers and stored at +4 °C until analysis. The median age of the participants was 54 years (aged 18–97); of them, 51% were female.

### 2.3 Extraction

All samples were extracted in duplicate: the first aliquot (replicate 1) was spiked with an IS mixture (2–10 ng) and used for target analysis; the second aliquot (replicate 2) was extracted without adding IS and was used for EOF analysis (see SI 3, Figure S2). The IPE was adapted due to its suitability for EOF extraction (low contamination with EOF).^38^ In brief, 2 mL of 0.5 M TBA solution in water and 5 mL of MTBE were added to the sample (replicate 1, 1.2 mL; replicate 2, 3 mL). The mixture was shaken horizontally for 15 min at 250 rpm and centrifuged for 10 min at 8500 rpm (8000 g) to separate the organic and aqueous phases. The MTBE layer was collected, and the extraction was repeated twice with 3 mL of MTBE. The extracts were combined and evaporated to 0.2 mL. The combined extracts were reconstituted to 1.0 mL with MeOH and evaporated down to 0.2 mL (replicate 1) and 0.5 mL (replicate 2). The replicate 1 sample extracts were split for instrumental analysis: most analytes were measured in the subsample with a 40% organic solvent content; the subsample with an 80% organic solvent content was used for PAPs and ultrashort-chain (C2–C3) PFAS analysis. A replicate 2 subsamples, pure extract, were analyzed for EOF content (details in SI 3 and Figure S3).

### 2.4 Instrumental Analysis and Quantification

The ultrashort-chain compounds (C2–C3) were measured using an SFC system (Waters Ultra Performance Convergence Chromatograph, UPCC; Waters Corporation, Milford) coupled to a XEVO TQ-S (Waters Corporation) tandem mass spectrometer (MS/MS) using CO_2_ and MeOH with 0.1% ammonia as mobile phases with a Torus DIOL analytical column (3.0 × 100 mm, 1.7 μm).^39^

The majority of target analytes (≥C4) were quantified using an Acquity UPLC coupled with a Xevo TQ-S MS/MS (both from Waters Corporation). Separation was achieved with a C18 BEH column (2.1 × 100 mm, 1.7 μm); mobile phases were MeOH and a 30/70 (v/v) mixture of MeOH and water with 2 mmol/L ammonium acetate and 5 mmol/L 1-methylpiperidine as additives.^40^ Two novel PFAS (ADONA and hexafluoropropylene oxide-dimer acid (HFPO-DA)) were measured using a Waters Acquity UPLC system coupled with a XEVO TQ-S micro MS/MS; the column and mobile phase were the same as shown before. Additional details on the multiple reaction monitoring (MRM) methods are in SI 5 Table S2.

In this study, concentrations of 63 PFAS were monitored; their calibration ranges were between 0.005 and 30 ng/mL. MRM was used to improve selectivity, and at least two transitions were monitored for most analytes; a single transition was monitored for TFA, PFPrA, perfluorobutanoic acid (PFBA), and perfluoropentanoic acid (PFPeA). The following PFOS isomers/isomer groups were quantified: 1-*m*-PFOS, 6/2-*m*-PFOS, 3/4/5-*m*-PFOS, and 4.4/4.5/5.5-*m*_2_-PFOS, and their concentration was reported as the sum of branched PFOS isomers. Concentrations of all analytes were corrected for recovery by using internal standards. Limits of quantification (LOQs) were determined based on several criteria: (i) the signal-to-noise ratio of the peak had to be more than 10, (ii) the lowest point of the calibration curve, and (iii) the procedural blank level plus 10 times the pooled standard deviation. More details are in SI 5 Table S3. Because the ultrashort-chain perfluoroalkyl carboxylic acid (PFCA) analysis was qualitative due to the lack of suitable internal standards, their levels were only included in the fluorine mass balance and profiles of sum PFAS in Section 3.2 are presented as a sum of 61 PFAS (∑_61_PFAS).

EOF levels were determined with a CIC system composed of a combustion module (Analytik Jena, Germany), a 920 Absorber Module, and a 930 Compact IC Flex ion chromatograph module (both from Metrohm, Switzerland). An ion-exchange column (Metrosep A Supp 5–150/4.0), with a carbonate buffer (64 mmol/L sodium carbonate and 20 mmol/L sodium bicarbonate) as the mobile phase, was used for the separation of anions. The absorber solution was water.

The EOF results were obtained using an external calibration curve (50–1000 ng/mL F) made from a solid PFOS potassium salt (Fluka, Hampton). As background contamination of fluoride was observed in the CIC system, this was determined separately for each sample and subtracted from samples before further data analysis. To ensure reliability, the analysis of samples commenced only once background levels were stable with a relative standard deviation (RSD) below 5% for three consecutive background signal measurements. The LOQ was determined separately for each sample preparation batch as the average of the procedural blank of the batch plus three times the standard deviation of the procedural blanks. The reported EOF values were not additionally corrected for procedural blanks.

### 2.5 Quality Assurance and Quality Control Measures

Every batch of samples included one procedural blank for target analysis to keep track of possible contamination with known PFAS and a second procedural blank to monitor for any contamination with EOF. Each extraction batch (*n* = 19) included a quality control (QC) sample (SRM1957) to monitor both accuracy and reproducibility (results in SI 8, Table S10).

The recoveries for each sample (in replicate 1 used for target analysis) were determined with the use of recovery standards (additional isotopically labeled standards added to the sample after extraction prior to instrumental analysis); the acceptable recovery range was from 20 to 150%. Analytes with recoveries outside of the set range were marked as not quantified (n.q.).

The combustion blanks (empty boat injection in CIC) were used to evaluate possible carryover between consecutive samples; repeated injections of standard solution (samples with known EOF contents) were used to monitor the performance of the system—these results had to be within 20% of their nominal value. The repeatability of the CIC system was tested by triplicate analysis of samples prepared from the multielement anion standard solution; the RSD was below 25%.

### 2.6 Data Treatment

When concentrations of analytes in target PFAS analysis were below LOQ, zero was assigned for them for any further data treatment. The sum concentration of the 63 PFASs (∑_63_PFAS) in samples was calculated by excluding all values below LOQ. Below LOQ and above LOD, results were only used when calculating detection frequencies (above LOQ + between LOD and LOQ). The fluorine mass balance analysis was performed only on samples with EOF concentrations above LOD. To compare target PFAS data with EOF results, the fluorine content from each analyte was calculated and summed up to obtain the amount of fluorine explained by known PFAS.

For fluorine mass balance analysis, the measured PFAS concentrations of all analytes (ng/mL PFAS) were converted to respective fluoride concentrations (ng/mL F) using the formula
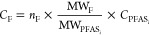
where *C*_F_ is the concentration of fluoride (ng/mL F) coming from the compound, *n*_F_ is the number of fluorine atoms in an analyte molecule, MW_F_ is the molecular weight of fluorine, MW_PFASi_ is the molecular weight of the analyte *i*, and *C*_PFASi_ is the concentration of analyte *i* (ng/mL PFAS *i*).

For statistical evaluation, the samples used for OF mass balance analysis were divided by age (group 1: ages 18–44 (*n* = 54); group 2: 45–70 (*n* = 53); and group 3: 71–97 (*n* = 41)) and further by gender. Three age groups were chosen to maintain a comparable sample size between the demographic groups. It was chosen not to group samples based on sampling location due to the uneven age distribution at the different sampling locations. The Kruskal–Wallis test was performed on the UOF percentage using the Real Statistics Resource Pack (release 7.7.2); the α value was set to 0.05.

## 3 Results

### 3.1 Organofluorine Mass Balance

**Figure 1:**
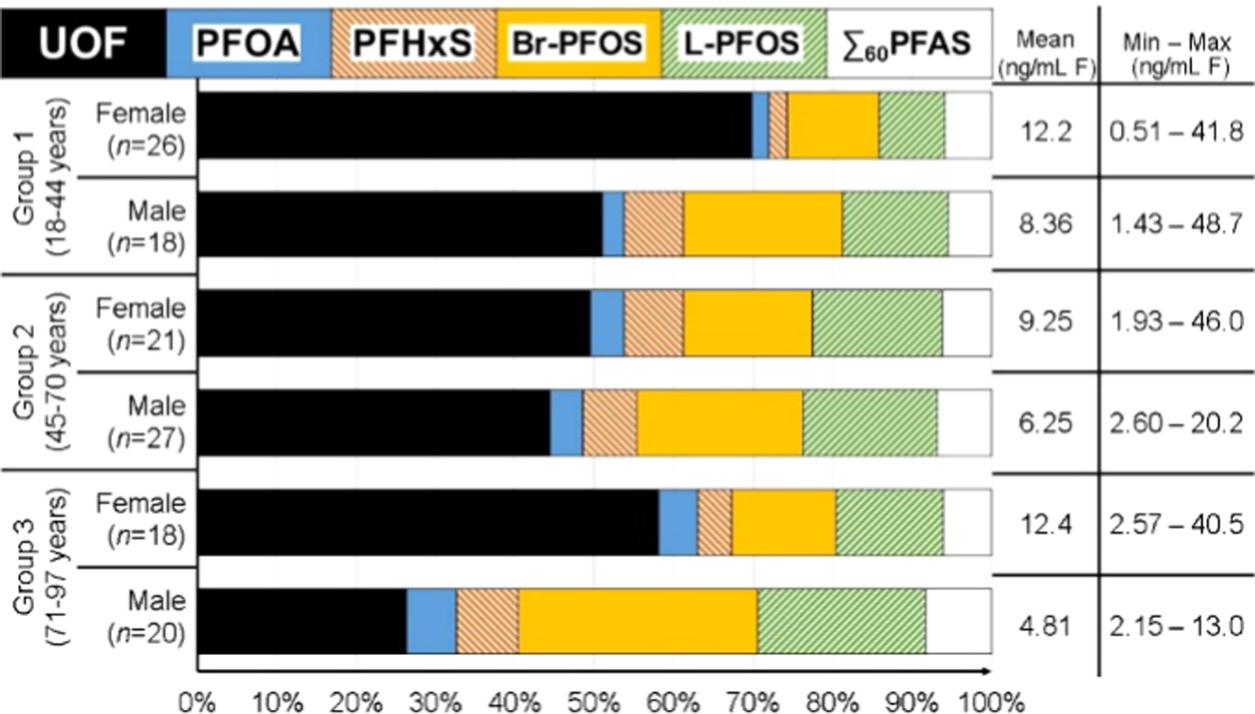
Organofluorine mass balance analysis as determined by EOF and target PFAS analysis in whole blood samples. The mass balance is made of unidentified organofluorine (UOF) and target PFAS. The values given are EOF concentrations (ng/mL F). ∑_60_PFAS, all measured PFAS with the exception of PFOA, PFHxS, and PFOS (linear + branched).

Organofluorine mass balance analysis was performed for 130 samples that had EOF levels above the LOD; samples below LOD (*n* = 17) were excluded from this analysis. The OF mass balance profiles and EOF concentrations are shown in Figure 1. The highest mean EOF concentrations were found in samples from females, group 3 (average EOF of 12.4 ng/mL F; aged 71–97) and group 1 (12.2 ng/mL F; aged 18–44). A total of 20 samples from different age groups had all of their EOF explained by the target analytes. A supplementary figure with the concentrations is provided in SI 6, Figure S4.

The greatest proportion of UOF was found in samples from group 1 (18–44 years) females (70%; see Figure 1). The target PFAS accounting for most of the identified EOF in these samples were branched and linear PFOS (Br- and L-PFOS), 12 and 8%, respectively. Samples of group 3 (71–97 years) females had a smaller fraction of UOF, on average 58%. The identified EOF fraction was driven by PFOS; branched and linear isomers together accounted for 27% of EOF. The fluorine mass balance profiles were similar for groups 1 and 2 males and group 2 females, where UOF accounted for between 44 and 51% of EOF. Br- and L-PFOS together accounted for between 33 and 38% of EOF in these sample groups. The smallest fraction of UOF was found in group 3 males, 26% of EOF (see Figure 1). The main drivers of EOF in those samples were PFOS, accounting for 51% of EOF (L- and Br-PFOS together).

**Figure 2:**
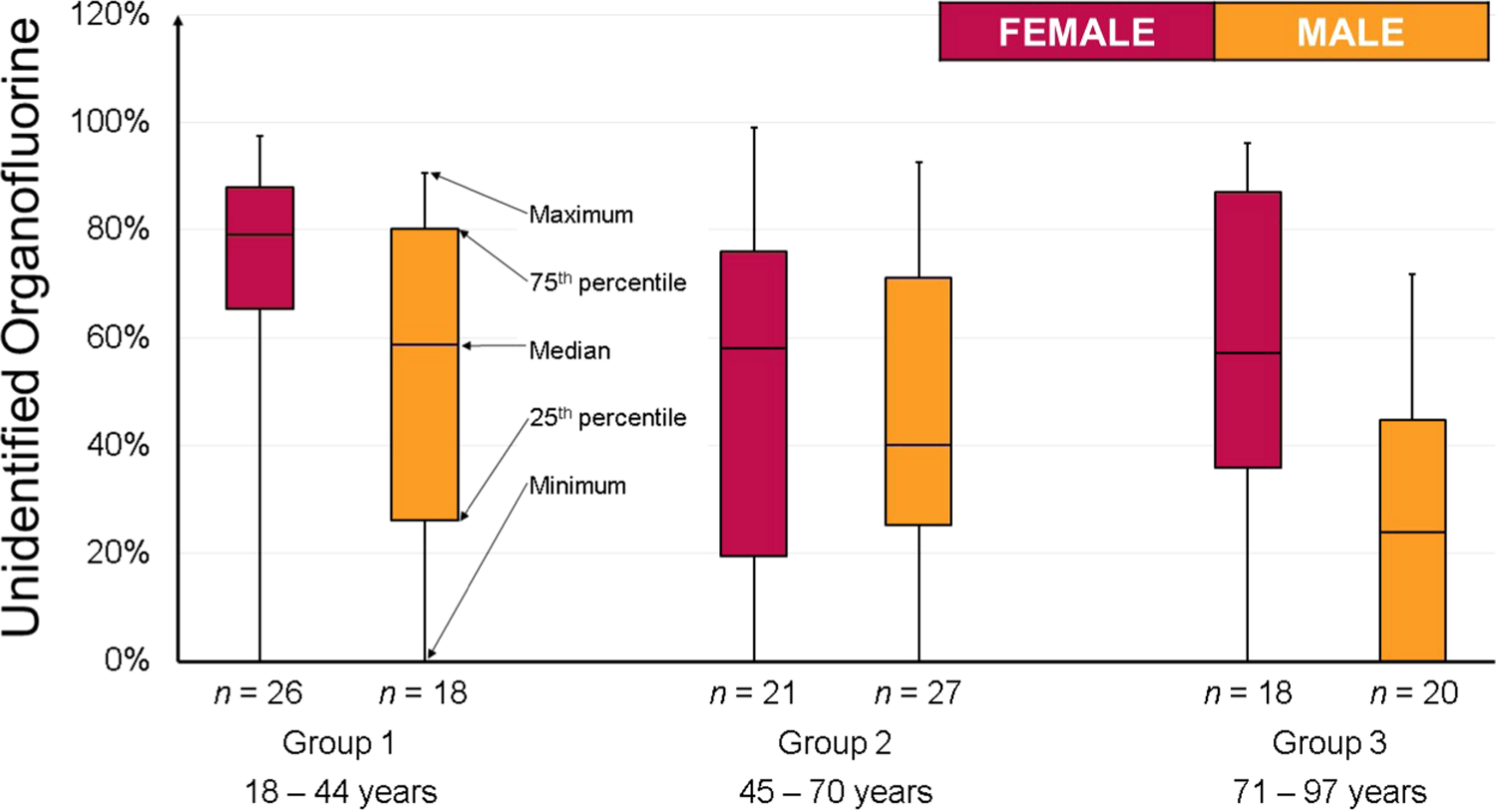
Boxplots of the percentage of UOF in different demographic groups.

Statistical analysis of the differences in UOF fractions between the demographic groups (total 130 samples, boxplots shown in Figure 2) was performed using the Kruskal–Wallis test since the results were not normally distributed. The first test between male (*n* = 65) and female (*n* = 65) samples revealed a statistically significant difference (*p* < 0.05) between the genders with females having a higher UOF fraction in their samples. This was followed by a Kruskal–Wallis test on the female samples divided into three age groups (*n* = 18), which revealed statistically significant differences (*p* < 0.05) between the female age groups; the youngest group of females had the highest fraction of UOF. Statistically significant differences (*p* < 0.05) were also found between male age groups (*n* = 18); the youngest group had the highest percentage of UOF.

### 3.2 Target PFAS

**Figure 3:**
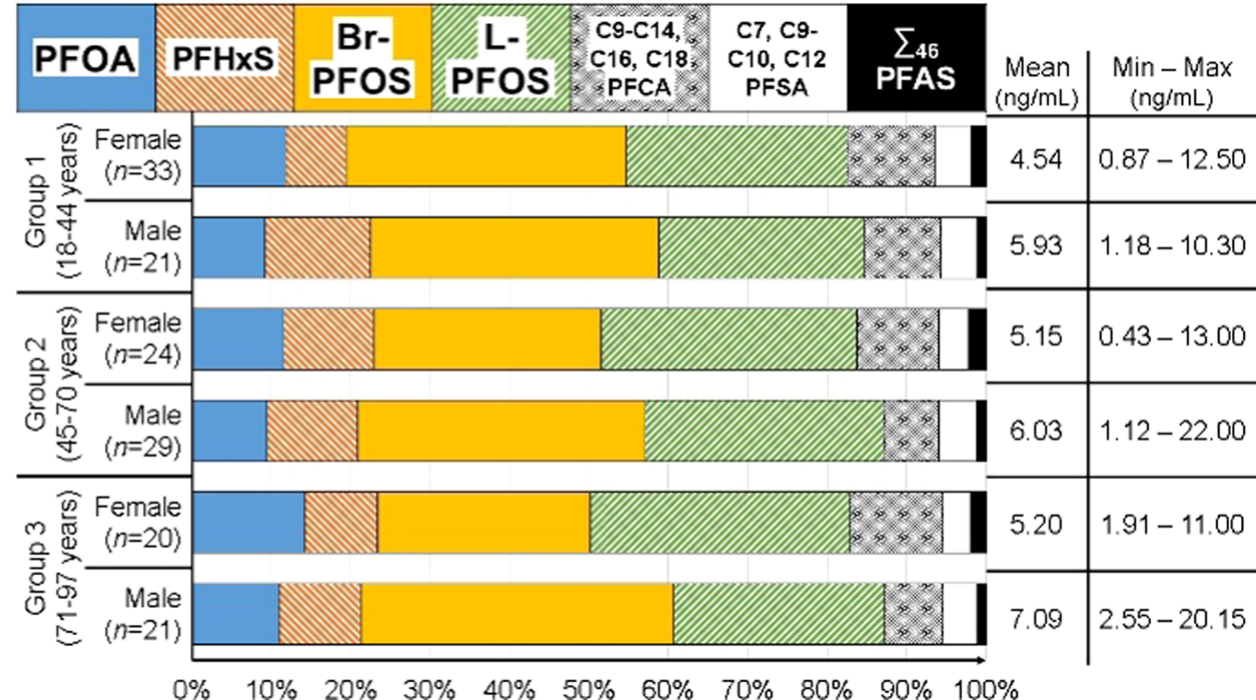
PFAS profile showing the contribution of the major PFAS, PFAS classes, and the remaining 46 PFAS to the ∑_61_PFAS levels (ng/mL) in the whole blood samples from Sweden along with ∑_61_PFAS levels (ng/mL).

The highest mean ∑_61_PFAS concentrations were found in whole blood samples from group 3 males (7.09 ng/mL). They were followed by group 1 and 2 males, whose mean ∑_61_PFAS concentrations were 5.93 and 6.03 ng/mL, respectively. The mean ∑_61_PFAS concentrations were slightly lower in group 2 and 3 females, 5.2 ng/mL in both groups. The lowest mean ∑_61_PFAS concentration was measured in group 1 females (4.54 ng/mL). The PFAS profiles are shown in Figure 3, and further details are shown in SI 6, Tables S4–S8. The ultrashort-chain PFCAs were excluded from the target analysis due to a lack of suitable mass-labeled standards, but the presence of TFA and PFPrA was detected in 62 and 22% of the samples, respectively. These compounds were only reported as detected or not detected in the target analysis.

Across all samples, the largest contributors to ∑_61_PFAS concentrations were Br- and L-PFOS, on average 34 and 29% of ∑_61_PFAS, respectively. The fraction of Br-PFOS ranged from 27% in group 3 females to 39% in group 3 males, while the percentage of ∑_61_PFAS accounted for by L-PFOS ranged from 26% in group 1 males to 33% in group 3 females. The ratio of Br-PFOS to sum PFOS (linear + branched) was the lowest in group 3 females (47% of sum PFOS was branched) and the highest in group 1 males (67% of sum PFOS). More details on the distribution of PFOS isomers in the demographic groups are given in SI 7, Table S9. The contribution of PFOA ranged from 9.3 to 14%, with an average of 11%. The average contribution of PFHxS was similar (10%); it ranged from 7.5% in group 1 females to 13% in group 1 males (see Figure 3 for details).

The remaining long-chain PFCAs accounted for anywhere between 6.9 and 12% of ∑_61_PFAS, most of which was attributable to C9–C11 PFCAs. Long-chain perfluoroalkyl sulfonic acids (PFSAs, excluding PFOS) made up a further 4% of ∑_61_PFAS on average and consisted exclusively of perfluoroheptane sulfonic acid (PFHpS).

The remaining 46 PFAS accounted for 1–2% of ∑_61_PFAS; perfluoroethyl-cyclohexane sulfonic acid (PFECHS) and ADONA were detected at trace levels in 80 and 16% of samples, respectively. Of the remaining 46 PFAS, PFSA precursor compounds contributed a further 0.4% of the ∑_61_PFAS and the remaining PFAAs (ultrashort-chain PFSAs, short-chain PFSAs, and PFCAs) contributed an additional 0.6%. The most commonly detected PFCA and PFSA precursors were 8:2 fluorotelomer sulfonic acid (8:2 FTSA, detected in 24% of samples) and methyl perfluorooctane sulfonamidoacetic acid (MeFOSAA in 16% of samples). The ultrashort-chain PFSAs, perfluoroethane sulfonic acid (PFEtS) and perfluoropropane sulfonic acid (PFPrS), were detected in 49 and 1% of the samples, respectively.

## 4 Discussion

To the best of our knowledge, this is the first study showing a statistically significant (*p* < 0.05) difference in the percentage of UOF between genders and age groups (see Figure 2), with females having a higher UOF fraction that was especially pronounced for the youngest females. Combined with females having consistently higher EOF concentrations (Figure 1), these results indicate that females have a higher UOF internal exposure (concentration) than males. This is contrary to what is generally observed for target PFAS where males have a higher target PFAS concentration (e.g., PFOS, PFOA) than females, which was also the case in this study (Figure 3).^41^

The likely sources of PFAS and UOF are personal care products as they are in contact with the skin and there is a risk of hand-to-mouth exposure.^42^ High concentrations of PFCAs (up to 9220 ng/g) and PAPs (up to 405 μg/g) have been found in personal care products in Sweden,^42^ and high PFAS concentrations in personal care products have been reported elsewhere as well.^43,44^ As PAPs are PFCA precursors, they could be metabolized and the intermediates would contribute to the UOF fraction. Dermal uptake of PFAS could be plausible as demonstrated by Franko and co-authors for PFOA.^45^ While both men and women use personal care products,^46^ the frequency of use is higher among women.^46,47^ This would suggest that if personal care products contribute to the OF mass balance, it would be more pronounced in females and could be one of the reasons behind females having higher EOF levels in this study (see Figure 1). The difference in exposure routes between genders could have been a contributing factor to the different ratios of Br-PFOS to ∑PFOS (SI 7, Table S9). For females, the fraction of L-PFOS (as part of ∑PFOS) increased with age, possibly due to its longer half-life than Br-PFOS isomers.^48^

Another potential source of OF in human blood samples could also be fluorinated pharmaceuticals. The number of fluorinated pharmaceuticals approved for human use has been increasing.^49,50^ For example, atorvastatin (C_33_H_35_FN_2_O_5_) was one of the most commonly prescribed drugs in Sweden in 2019.^51^ To which degree fluorinated pharmaceuticals contribute to the UOF fraction is not known, but atorvastatin can reach a blood concentration of 22 ng/mL (0.8 ng/mL F),^52^ which would account for approximately 10% of EOF in the samples measured in this study. The extraction solvent used in this study (MTBE) has been used in liquid–liquid extraction methods to extract atorvastatin from plasma samples.^53^ While the IPE method may not be optimized for it, this compound is likely to be coextracted at least a portion of atorvastatin or other fluorinated pharmaceuticals. The samples were collected from volunteers, and it is possible that some of them may have been taking fluorinated pharmaceuticals. This could contribute to the variability of the EOF concentrations in the demographic groups but is unlikely to impact the overall observation of the high UOF fraction due to short half-lives.^54,55^

A recent publication by Poothong and co-authors estimated the exposure routes for different PFAS, including PFOA and PFOS; around 90% was from ingestion, and the remainder was through house dust, indoor air, and dermal absorption.^56^ Indoor dust has been identified as an exposure route for PFAS,^56^ and it is likely to be a UOF exposure route as well, although to which extent is still unknown. Plastic additives have already been reported in indoor dust;^57^ thus, there would be reason to expect fluorinated additives (e.g., fluoro-modified acrylic polymers) in dust as well.^58,59^ While the concentrations of these additives may be between 0.01 and 1.0% in a given plastic item, the global plastics production reached 368 million tonnes by 2019.^60^ Indoor dust has been reported to contain fluorinated liquid crystal monomers (LCMs), and these compounds could represent an additional source of UOF through dust inhalation since 48% of the currently produced LCMs contain fluorine.^61^ Fluorinated LCMs have been reported in dust from e-waste dust^62^ and in sediment samples from China,^63^ indicating environmental presence and potential for additional human exposure.

Given the presence of both precursor compounds (e.g., 8:2 FTSA and MeFOSAA) and stable degradation end products (PFAAs) in human blood,^34,37^ it is likely that the samples also contained degradation intermediates that were not measured in this study. These intermediate products would contribute to both the overall EOF concentration and specifically the UOF fraction because many of these intermediates were not included in the target analysis. It is unclear to what degree the precursors degrade to stable PFAAs and how large of a proportion remains in the human body as intermediates.

**Figure 4:**
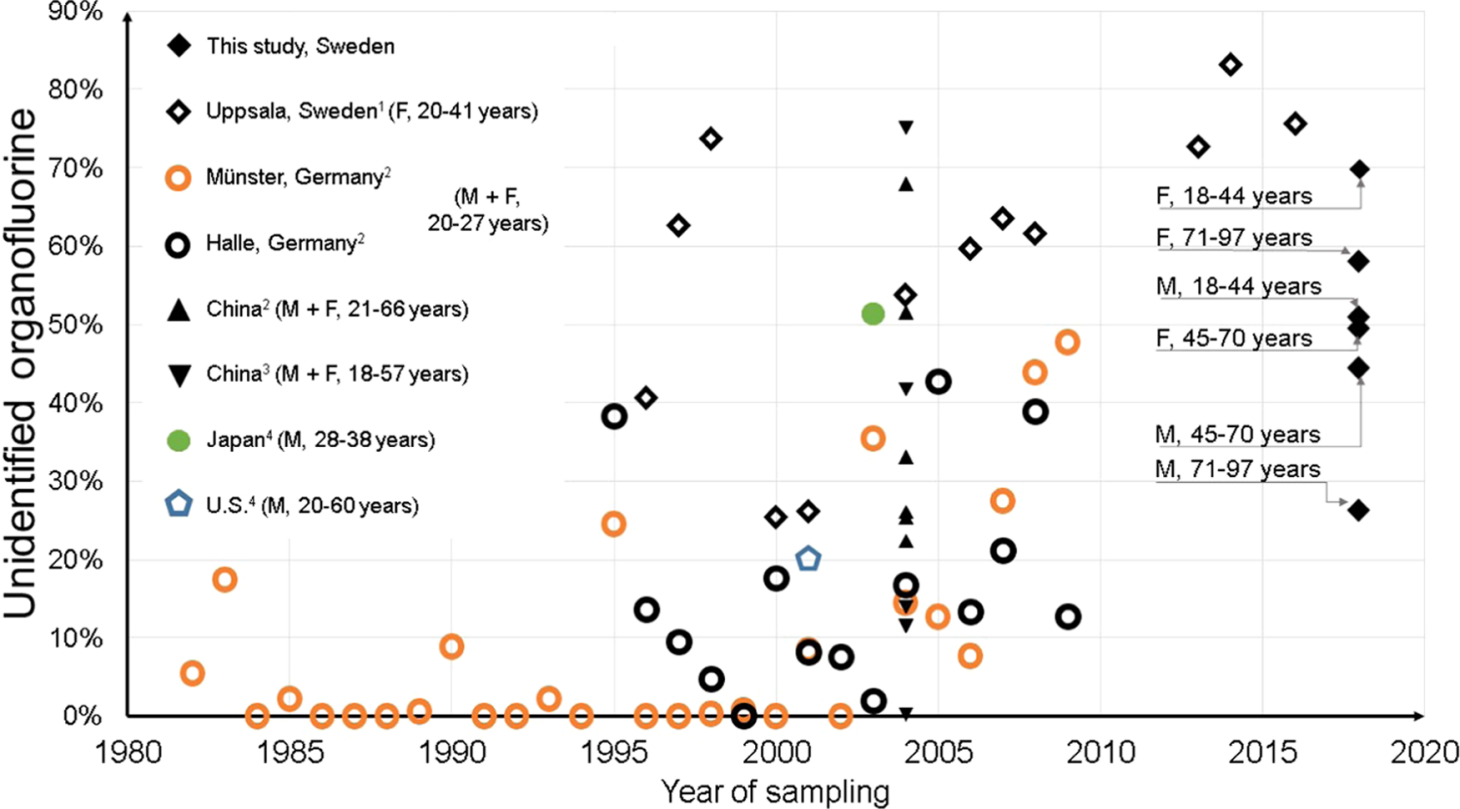
Fraction of unidentified organofluorine (UOF) measured in human blood, plasma, and serum samples in this and past studies. M, male; F, female. Filled marker, whole blood; unfilled marker, plasma/serum. ^1^Miaz 2020, ^2^Yeung 2016, ^3^Yeung 2008, ^4^Miyake 2007.

The results from this study are in line with the previous work investigating the fluorine mass balance in human whole blood, serum, and plasma from the United States, Japan, China, Germany, and Sweden since 2007.^32−34,37^ In combination, these publications indicate that the fraction of UOF has been increasing (see Figure 4), despite overall reductions in PFOS and PFOA levels.^34,37^ Comparing fluorine mass balance studies can be challenging due to the use of different sample matrices (plasma^37^ or whole blood^32^). Some unidentified compounds could potentially partition preferably to one of the matrices (e.g., plasma) over the others, and it has been studied for target analytes^64^ but not for EOF. An additional source of bias may be the chosen extraction method, as every extraction method will extract a different fraction of the OF compounds present in the sample. Of the EOF studies listed above, the IPE was the most common one^32−34^ and also used in this study, while Miaz et al. made use of a protein precipitation.^37^

In this study, 26 individual whole blood samples from females aged 18–44 were collected across Sweden in 2018–2019 and 70% of the EOF in those samples was unidentified. Miaz et al. analyzed three pooled serum samples (females aged 24–36, each a pool of 10 samples) collected in 2016 from Uppsala and extracted the samples using an acetonitrile method and found a mean UOF fraction of 76%.^37^ This indicates that the OF mass balance analysis of whole blood and serum samples is comparable to each other; the fraction of UOF remains similar in both matrices. While using serum or plasma samples could exclude some PFAS from the OF mass balance analysis,^64^ their concentrations seem not to have a large impact on the OF mass balance. This suggests that both serum and plasma are suitable for future studies investigating the OF mass balance in human samples.

The EOF levels in this study and that of Miaz et al. were also comparable, 12.2 ng/mL F (youngest group of females) and 14.2 ng/mL F (2016 results after conversion to whole blood basis), respectively.^37^ The variability of the EOF results in this study is in line with previous studies; for example, Yeung and Mabury measured the EOF content of 34 whole blood samples from China^34^ and had a relative standard deviation of the mean of 13%, while the relative standard deviation of the mean was 10% in this study (*n* = 130).

**Figure 5:**
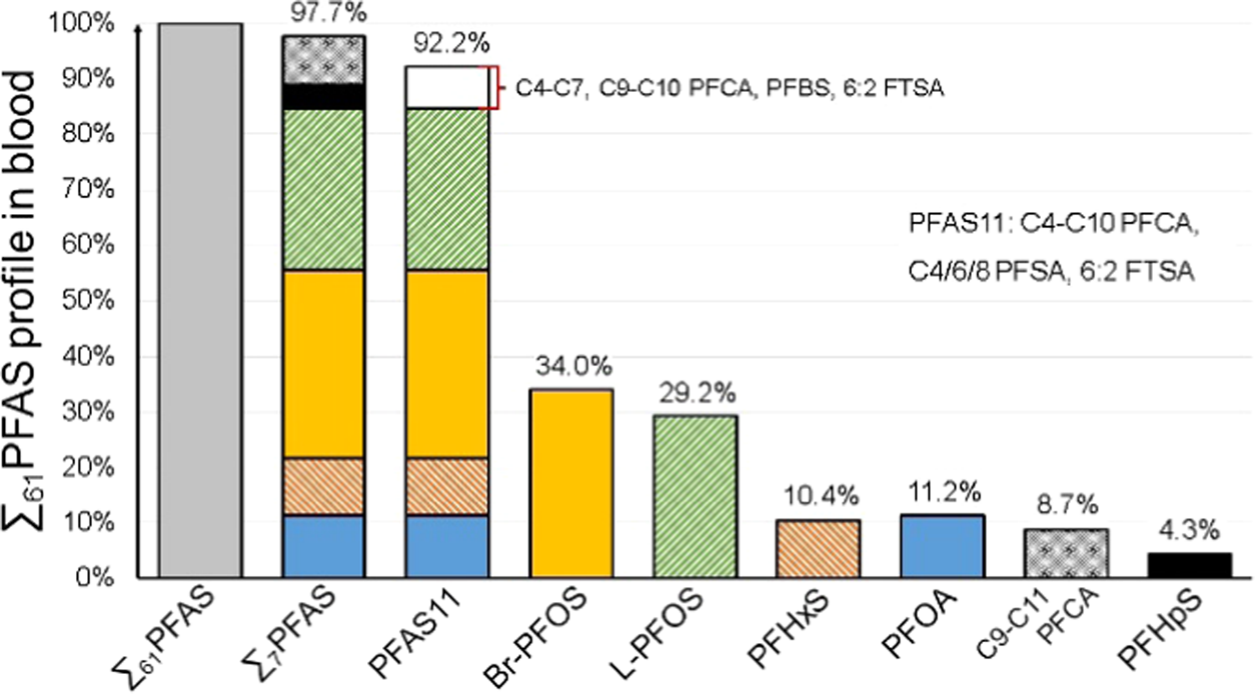
Contribution of different PFAS to the ∑_61_PFAS profile in human blood samples from Sweden.

In light of the thousands of potential analytes,^20^ it is critical for biomonitoring studies to select the most relevant analytes to ensure the greatest analytical coverage possible within time and budget restraints. While EOF analysis would provide wider analytical coverage, the large sample amount required (due to high LODs) limits its wider adoption at this time. This leaves target analysis, for example, using UPLC-MS/MS, as a viable methodology. The results from this study indicate that a limited number of analytes, e.g., PFAS11 of the Swedish Food Agency,^65^ can be sufficient to account for over 90% of the ∑_61_PFAS. The seven PFAS with the highest contribution to the ∑_61_PFAS in blood samples (Br- and L-PFOS, PFHxS, PFOA, PFHpS, PFNA, PFDA, and PFUnDA, together accounting for 98% of the ∑_61_PFAS on average) are shown in Figure 5. The remaining 48 analytes included in this study had a negligible impact on the ∑_61_PFAS. These results highlight the importance of choosing target PFAS relevant for the study matrix, e.g., branched PFOS isomers for human biomonitoring, as they accounted for 35% of the ∑_61_PFAS. Excluding branched PFOS isomers would result in an underestimation of the PFAS levels in humans and thus potentially underestimating the health risks.

Relatively high detection frequencies of some of the ultrashort-chain PFAAs (e.g., TFA: 62%, PFPrA: 22%, PFEtS: 49%) to other short-chain PFAS (C4–C6 PFCAs (below 10% detection frequency)) were observed in this investigation. Despite their short half-lives,^66^ the results suggest continuous exposure to these compounds in the environment, drinking water, or through the metabolism of precursor compounds. To the best of our knowledge, this is the first study reporting PFEtS in human blood, signaling potential exposure to contamination from aqueous film-forming foams.^67^ TFA and PFPrA have been found with a high detection frequency in the Chinese general population as well, but that study excluded PFEtS and PFPrS.^68^ It would be important to understand the exposure route and effects of these ultrashort-chain PFAAs in future studies.

Another highlight of this study was the detection of PFECHS in 80% of all of the samples (details in SI 6 Table S8). PFECHS was also recently found at low concentrations in human serum samples from Uppsala (Sweden).^37^ While this compound has been detected in various environmental samples before (biota,^69^ surface water,^70^ and sediment^71^), this is the first time that its widespread occurrence in the general population has been found. Initially, it was suggested that one of the possible sources of PFECHS could be the aviation industry, where it is used as an erosion inhibitor,^70^ but additional uses have been identified.^72,73^

The PFOS levels found in the Swedish general population in this study (3.6 ng/mL, sum of linear and branched PFOS) were similar to PFOS levels elsewhere in the world (converted to whole blood concentrations): 2.4 ng/mL in the United States,^74^ 1.4 ng/mL in Germany,^75^ and 7.2 ng/mL in China.^76^ Nevertheless, this is almost a decade after PFOS and its salts were added to Annex B of the Stockholm Convention, marking them up for restrictions in production and use^77^ and additional regulations later on.^78^ While older studies showed a rapid decrease in the concentrations of PFOS^36,79^ and PFOA^35^ in human plasma samples, the rate of this decrease seems to have slowed down.^37^ Given that the half-life of PFOS in humans is 5.4 years,^80^ the continued presence of PFOS in human samples hints at additional exposure sources, potentially through recirculation.

Exposure to PFAA precursors is confirmed by the detection of PFAA precursor compounds in this study. One method to better understand the PFAA precursor levels would be to use the TOP assay, but several questions remain unanswered for this method. Should the whole sample (in this case blood) be oxidized, then optimizing the reagent amounts would be challenging. An alternative would be to perform the TOP assay on the sample extract, but only the compounds that can be extracted from the sample will undergo oxidation. This is a limitation shared with EOF analysis, but in the case of the TOP assay, it is also necessary for the precursor compounds to be oxidizable to readily measurable PFAAs.

Another path to close the OF mass balance would be to expand the number of measured PFAS. However, the inclusion of a few additional compounds would have a low impact since the concentrations would be very likely low, e.g., PFECHS in this study. This approach could have more merit close to known point sources, for example, fluoropolymer production sites. Comprehensive monitoring of the relevant environmental matrices (air, water) should raise an alarm well in advance and the human biomonitoring programs could be modified. The inclusion of fluorinated pharmaceuticals is likely to yield minimal gains in the OF mass balance due to their rapid elimination and it may be more relevant for municipal sewage studies, as this is where they would end up after use, before being released back into the environment.

The measurement of EOF allows the analyst to rapidly identify cases of high contamination in need of further investigation. With a single measurement, it is possible to estimate whether the individual sample is an outlier from the general population. However, for wider adoption of the EOF approach in human biomonitoring, the sensitivity of the CIC analysis would need to be improved by a factor of 10 (permitting smaller sample volumes; this study required 3 mL of whole blood). If a sample has a high EOF content, then further investigation of the sample and later identifying the exposure sources (e.g., occupational exposure) could be undertaken. However, the work toward identifying novel PFAS should be pursued as well. There have been instances where the replacement product, of, for example, PFOS, ends up being just as toxic.^81^ The UOF fraction could be composed of a single organofluorine compound at high concentrations or several compounds at low concentrations, but given the endocrine disruptive potential of PFAS,^6,7^ even very low concentrations may have an adverse health effect; any amount of UOF is a potential health hazard. Until the constituents are identified, it is not possible to assess the risk, which in turn may lead to misguided policy decisions, for example, in environmental regulations.

## Supporting Information Available

It provides details of sampling locations and extraction methods; list of target analytes and respective mass spectrometer parameters; and results for different demographic groups from target PFAS analysis along with the ratios of PFOS isomers (PDF)
