# Supplementary material for: Organofluorine Mass Balance Analysis of Whole Blood Samples in Relation to Gender and Age
Source: Environ Sci Technol. 2021 Sep 14;55(19):13142–51. doi: 10.1021/acs.est.1c04031 (PMC8495894; doi:10.1021/acs.est.1c04031)
Supplement: Supplementary file 1 — es1c04031_si_001.pdf [file es1c04031_si_001.pdf]

Supporting Information

Organofluorine Mass Balance Analysis of Whole Blood Samples in Relation to Gender and Age

Rudolf Aro, Ulrika Eriksson, Anna Kärrman, Leo W.Y. Yeung*

Man-Technology-Environment (MTM) Research Centre, School of Science and Technology, Örebro University, Sweden, SE-701 82

*Corresponding author

E-mail: leo.yeung@oru.se

Summary

Number of Pages: 17

Supplemental Figures: 4

Supplemental Tables: 10

Table of Contents

1. Chemicals S3
2. Sampling Locations S3
3. Sample Extraction S3
4. List of Analytes S6
5. Mass Spectrometer Parameters S8
6. Results of PFAS Analysis S10
7. Ratio of PFOS Isomers S16
8. Results from NIST SRM1957 .... S16
9. Bibliography S17

1. Chemicals

Methanol (MeOH) for extractions and mobile phases was purchased from Fisher Scientific (Hampton, United States) and the additives for ultra performance liquid chromatography (UPLC) mobile phases (ammonium acetate and 1-methylpiperidine) were purchased from Sigma-Aldrich (Saint Louis, United States). Water used for extractions and mobile phases was purified with a MilliQ system to a resistance of 18.2 MΩ. Additional chemicals for sample extraction, tetrabutyl-ammonium sulfate (TBA) and methyl tert-butyl ether (MTBE), were purchased from Sigma-Aldrich. For supercritical fluid chromatography (SFC) the eluent additive, ammonia, and mobile phase, CO_2, were purchased from Fisher Scientific and AGA (Lidingö, Sweden), respectively.

The standard for ion chromatography, multielement anion standard solution (product nr: 89886), was purchased from Sigma-Aldrich. The standard reference material for sample extraction, SRM1957 (Organic Contaminants in Non-Fortified Human Serum), was purchased from the National Institute of Standards and Technology (NIST).

2. Sampling Locations

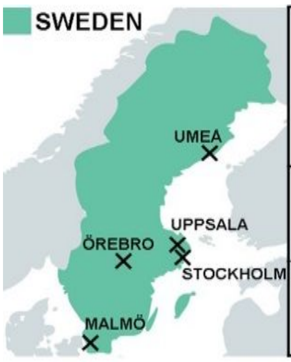

	Group 1 18 – 44 years		Group 2 45 – 70 years		Group 3 71 – 97 years	
	Female	Male	Female	Male	Female	Male
n for EOF analysis (*n*=130)	26	18	21	27	18	20
n for PFAS analysis (*n*=148)	33	21	24	29	20	21

Figure S1. Sampling locations and the demographic information.

3. Sample Extraction

All samples were extracted in duplicate (see Figure S2), the first one (Replicate 1) was spiked with internal standards (IS) before the extraction and used for target analysis (Figure S2). The second replicate (Replicate 2) was extracted without spiking any IS and analyzed for EOF content.

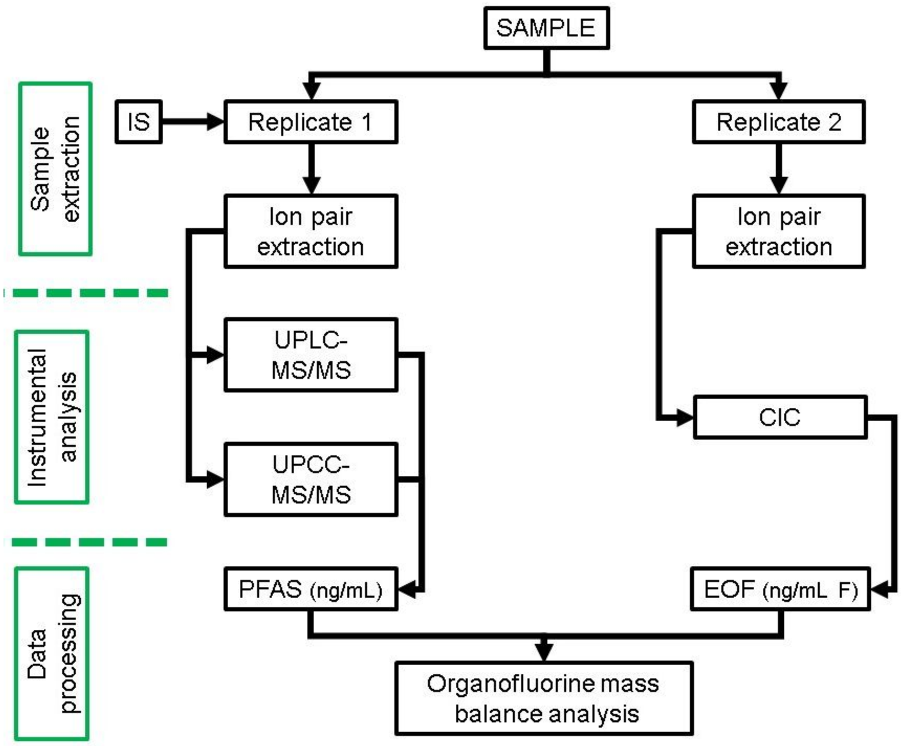

Figure S2. Schematic representation of the workflow in this study.

Prior to sample extraction, individual whole blood samples were vigorously shaken and/or vortexed to mix the contents of each vacutainer. Two aliquots of the whole blood were taken into pre-cleaned 15 mL polypropylene (PP) tubes (rinsed three times with methanol (MeOH) and allowed to dry), the mass of each sub-sample was recorded. The subsample for target analysis (Replicate 1) was spiked with an IS mixture (10 µL, 2 ng of each IS apart from monoPAPs and HFPO-DA of which 5 ng were added); the second subsample, for EOF analysis (Replicate 2), was extracted without any IS. The omission of IS for Replicate 2 was necessary as this would interfere with the EOF analysis, because the CIC system cannot differentiate between different sources of fluorine. These duplicate samples were extracted in the same batch to minimize the variability between them.

Samples were extracted in duplicates using the ion pair method [1]. In brief, 2 mL of 0.5 M tetrabutyl-ammonium (TBA) solution in water was added to the extract. Then, 5 mL of methyl tert-butyl ether (MTBE) was added to the tube. The mixture was shaken horizontally for 15 minutes at 250 rpm and centrifuged for 10 minutes at 8000 g to separate the organic and aqueous phases. The top layer (MTBE) was transferred to a new pre-cleaned PP tube and the extraction was repeated twice with 3 mL of MTBE. The extracts were combined and evaporated to 200 µL using an evaporation system. The combined extracts were reconstituted to 1.0 mL with MeOH and evaporated 0.2 mL (replicate 1) and 0.5 mL (replicate 2) with the evaporation system and the supernatants were transferred to LC vials. Most of the analytes were quantified in the sample with 40% organic solvent content. The sample with 80% organic

solvent content was used for PAPs and ultra-short chain PFAS analyses. Details on how the samples were split for instrumental are shown in Figure S3.

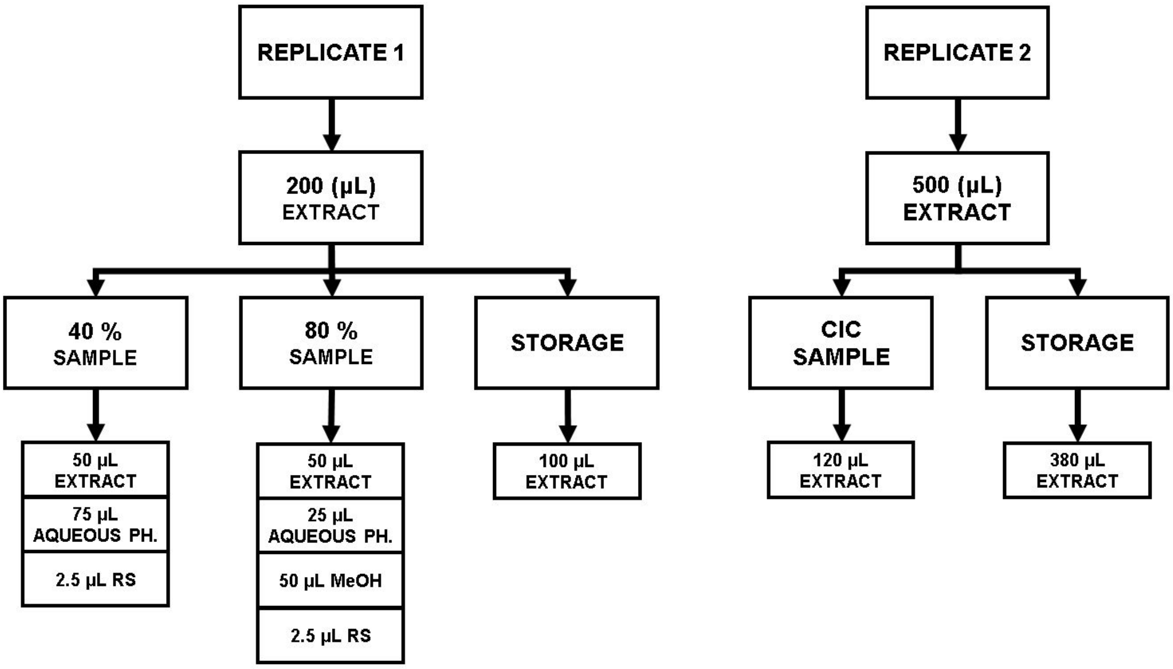

Figure S3. Detailed scheme of how the samples were divided for different instrumental analysis. Replicate 1 was analyzed with two different methanol compositions, 40% and 80% to improve chromatography. Replicate 2 was analyzed for the EOF content with CIC. RS – mass labelled recovery standard; aqueous phase – 2 mmol/L ammonia acetate in MilliQ water; all extracts were in methanol (MeOH).

4. List of Analytes

Table S1. List of abbreviations of target PFASs in this study.

Class	Subgroup	Acronym	Name
PFSA	Ultra-short chain	PFEtS	Perfluoroethane sulfonic acid
	Ultra-short chain	PFPrS	Perfluoropropane sulfonic acid
	Short-chain	PFBS	Perfluorobutane sulfonic acid
	Short-chain	PFPeS	Perfluoropentane sulfonic acid
	Long-chain	PFHxS	Perflurohexane sulfonic acid
	Long-chain	PFHpS	Perfluoroheptane sulfonic acid
	Long-chain	PFOS	Perfluorooctane sulfonic acid
	Long-chain	PFNS	Perfluorononane sulfonic acid
	Long-chain	PFDS	Perfluorodecane sulfonic acid
	Long-chain	PFDoDS	Perfluorododecane sulfonic acid
PFCA	Ultra-short chain	TFA	Trifluoroacetic acid
	Ultra-short chain	PFPrA	Perfluoropropanoic acid
	Short-chain	PFBA	Perfluorobutanoic acid
	Short-chain	PFPeA	Perfluoropentanoic acid
	Short-chain	PFHxA	Perfluorohexanoic acid
	Short-chain	PFHpA	Perfluoroheptanoic acid
	Long-chain	PFOA	Perfluorooctanoic acid
	Long-chain	PFNA	Perfluorononanoic acid
	Long-chain	PFDA	Perfluorodecanoic acid
	Long-chain	PFUnDA	Perfluoroundecanoic acid
	Long-chain	PFDoDA	Perfluorododecanoic acid
	Long-chain	PFTrDA	Perfluorotridecanoic acid
	Long-chain	PFTDA	Perfluorotetradecanoic acid
	Long-chain	PFHxDA	Perfluorohexadecanoic acid
	Long-chain	PFOcDA	Perfluorooctadecanoic acid
FTCA	Intermediate	3:3 FTCA	3:3 Fluorotelomer carboxylic acid
	Intermediate	5:3 FTCA	5:3 Fluorotelomer carboxylic acid
	Intermediate	7:3 FTCA	7:3 Fluorotelomer carboxylic acid
FTUCA	Precursor	6:2 FTUCA	6:2 Fluorotelomer unsaturated carboxylic acids
	Precursor	8:2 FTUCA	8:2 Fluorotelomer unsaturated carboxylic acids
	Precursor	10:2 FTUCA	10:2 Fluorotelomer unsaturated carboxylic acids
FTSA	Precursor	4:2 FTSA	4:2 Fluorotelomer sulfonic acid
	Precursor	6:2 FTSA	6:2 Fluorotelomer sulfonic acid
	Precursor	8:2 FTSA	8:2 Fluorotelomer sulfonic acid
	Precursor	10:2 FTSA	10:2 Fluorotelomer sulfonic acid
monoPAP	Precursor	6:2 monoPAP	6:2 Fluorotelomer phosphate monoester
	Precursor	8:2 monoPAP	8:2 Fluorotelomer phosphate monoester
	Precursor	10:2 monoPAP	10:2 Fluorotelomer phosphate monoester
diPAP	Precursor	6:2 diPAP	6:2 Fluorotelomer phosphate diester
	Precursor	8:2 diPAP	8:2 Fluorotelomer phosphate diester
	Precursor	6:2/8:2 diPAP	6:2/8:2 Fluorotelomer phosphate diester
	Precursor	10:2 diPAP	10:2 Fluorotelomer phosphate diester
PFPA		PFHxPA	Perfluorohexyl phosphonic acid
		PFOPA	Perfluorooctyl phosphonic acid
		PFDPA	Perfluorodecyl phosphonic acid
PFPiA	Potential precursors	C6/C6 PFPiA	Bis (perfluorohexyl) phosphinic acid
		C6/C8 PFPiA	Perfluoro (hexyloctyl) phosphinic acid
		C8/C8 PFPiA	Bis (perfluorooctyl) phosphinic acid
FASA	Precursor	FBSA	Perfluorobutane sulfonamide
		MeFBSA	Methyl perfluorobutane sulfonamide
		PFHxSA	Perfluorohexane sulfonamide
		MeFHxSA	Methyl perfluorohexane sulfonamide
		FOSA	Perfluorooctane sulfonamide
FASAA	Precursor	FOSAA	Perfluorooctane sulfonamidoacetic acid
	Precursor	MeFOSAA	Methyl perfluorooctane sulfonamidoacetic acid

	Precursor	EtFOSAA	Ethyl perfluorooctane sulfonamidoacetic acid
PFCHS	Novel	PFECHS	Perfluoroethylcyclohexane sulfonic acid
PFECA	Novel	ADONA	3H-perfluoro-3-[(3-methoxy-propoxy)propanoic acid]
	Novel	HFPO-DA (GenX)	Hexafluoropropylene oxide dimer acid
PFESA	Novel	6:2 Cl-PFESA (F-53B)	6:2 chlorinated polyfluorinated ether sulfonate
	Novel	8:2 Cl-PFESA	8:2 chlorinated polyfluorinated ether sulfonate

5. Mass Spectrometer Parameters

Table S2. List of analytes, MRM transitions, cone voltage, and collision energy used for quantification and qualification of PFAS.

Analyte	Precursor/ product ions quantification (m/z)	Cone (V)	Coll (eV)	Precursor/ product ions qualification (m/z)	Cone (V)	Coll (eV)	Internal standard
TFA	112.9/68.96	26	10				^{13}C-PFBA
PFPrA	162.97/118.9	20	10				^{13}C-PFBA
PFBA	212.97/169	20	11				^{13}C-PFBA
PFPeA	262.97/219	20	8				^{13}C-PFPeA
PFHxA	312.97/269	20	9	312.97/118.95	20	26	^{13}C-PFHxA
PFHpA	362.97/319	20	10	362.97/168.97	20	16	^{13}C-PFHpA
PFOA	412.97/369	20	10	412.97/168.97	20	18	^{13}C-PFOA
PFNA	462.99/419	20	12	462.99/219	20	18	^{13}C-PFNA
PFDA	512.97/469	20	11	512.97/219	20	18	^{13}C-PFDA
PFUnDA	562.97/519	20	12	562.97/268.99	20	18	^{13}C-PFUnDA
PFDoDA	612.97/569	34	14	612.97/168.96	40	22	^{13}C-PFUnDA
PFTrDA	662.9/619	20	14	662.9/168.96	20	26	^{13}C-PFUnDA
PFTDA	712.9/669	20	14	712.9/168.97	20	28	^{13}C-PFUnDA
PFHxDA	812.9/769	30	15	812.9/168.96	42	32	^{13}C-PFUnDA
PFOcDA	912.9/869	36	15	912.9/168.96	36	36	^{13}C-PFUnDA
PFEtS	198.8/79.8	65	20				^{13}C-PFBS
PFPrS	248.9/80.0	70	25				^{13}C-PFBS
PFBS	298.9/98.9	20	26	298.9/79.96	20	26	^{13}C-PFBS
PFPeS	348.90/98.96	20	26	348.90/79.96	20	30	^{13}C-PFHxS
PFHxS	398.9/98.9	20	30	398.9/79.96	20	34	^{18}O-PFHxS
PFHpS	448.97/98.90	20	30	448.97/79.96	20	35	^{13}C-PFOS
PFOS	498.97/98.96	20	38	498.97/79.96, 498.97/169.03	20	44, 34	^{13}C-PFOS
PFNS	548.90/98.96	20	38	548.90/79.96	20	44	^{13}C-PFOS
PFDS	598.97/98.9	20	42	598.97/79.96	20	58	^{13}C-PFOS
PFDoDS	698.90/98.90	20	40	698.90/79.96	20	45	^{13}C-PFOS
3:3 FTCA	240.9/136.97	10	16	240.9/116.93	10	22	^{13}C-PFPeA
5:3 FTCA	340.9/236.97	10	16	340.9/216.93	10	22	^{13}C-PFHpA
6:2 FTUCA	356.9/292.91	10	18	356.9/242.95	10	36	^{13}C-PFHpA
7:3 FTCA	440.9/336.89	12	14	440.9/316.93	12	20	^{13}C-PFNA
8:2 FTUCA	456.9/392.84	10	18	456.9/392.84	10	38	^{13}C-PFNA
10:2 FTUCA	556.84/492.82	8	16	556.84/242.94	8	38	^{13}C-PFUnDA
FBSA	297.9/77.92	20	20	297.9/118.94	20	15	^{13}C-PFHxA
MeFBSA	311.97/111.93	14	20	397.9/168.94	14	16	^{13}C-PFOA
PFHxSA	397.9/77.92	30	26	411.97/318.96	30	28	^{13}C-PFOS
MeFHxSA	411.97/168.93	24	24	411.97/318.96	24	20	^{13}C-PFOA
FOSA	497.9/78	82	30	497.9/168.96	82	29	^{13}C-PFOA
FOSAA				555.8/418.85			^{2}H -Et-FOSAA
MeFOSAA				569.78/482.76			^{2}H -Et-FOSAA
EtFOSAA				583.84/482.8			^{2}H -Et-FOSAA
4:2 FTSA	327/307	20	20	327/81	20	28	^{13}C-PFHxA
6:2 FTSA	427/407	20	20	427/81	20	28	^{13}C-PFOA
8:2 FTSA	527/507	20	20	527/80	20	28	^{13}C-PFDA
10:2 FTSA	627/607	20	20	627/80	20	28	^{13}C-PFUnDA
6:2 Cl-PFESA	530.9/351	58	24	530.9/83.0	58	24	^{13}C-PFOS
8:2 Cl-PFESA	630.9/451	58	24	630.9/83.0	58	24	^{13}C-PFOS
PFECHS	460.84/380.9	2	24	460.84/98.88	2	26	^{13}C-PFOA
6:2 mPAP	442.9/96.95			442.9/78.92			^{13}C-6:2mPAP
8:2 mPAP	542.9/97			542,9/78.92			^{13}C-8:2 mPAP
10:2 mPAP	642.968/97.005			642.968/78.98			^{13}C-8:2 mPAP
6:2 diPAP	788.9/97	64	28	788.9/442.91	64	18	^{13}C-6:2 diPAP

6:2/8:2 diPAP	888.78/96.94	66	34	888.78/442.81, 888.78/542.81	66	26	^{13}C-6:2 diPAP
8:2 diPAP	988.78/96.94	68	34	988.78/542.81	68	26	^{13}C-8:2 diPAP
10:2 diPAP	1188.78/96.94	68	34	1188.78/642.81	68	26	^{13}C-8:2 diPAP
SAmPAP	649.78/525.835			649.78/96.87			^{13}C-8:2 mPAP
diSAmPAP	1202.649/525.84			1202.649/168.902			^{13}C-8:2 diPAP
PFHxPA	398.97/79	62	26				^{13}C-PFOA
PFOPA	499/79	62	30				^{13}C-PFOA
PFDPA	599.03/79	62	30				^{13}C-PFNA
C6/C6 PFPiA	701/401	62	28				^{13}C-PFUnDA
C6/C8 PFPiA	801/401	24	28	801/501	24	28	^{13}C-PFUnDA
C8/C8 PFPiA	901/501	24	28				^{13}C-PFUnDA
HFPO-DA (GenX)	284.92/168.72	20	7	328.95/284.86	20	17	^{13}C-HFPO-DA
ADONA	376.97/250.8	30	37	376.97/84.69	15	29	^{13}C-HFPO-DA

Table S3. List of analytes and their minimum and maximum LODs, it was estimated for each sample preparation batch separately.

**Analyte**	**LOD min (pg/mL)**	**LOD max (pg/mL)**
PFBA	52	222
PFPeA	23	38
PFHxA	52	78
PFHpA	38	65
PFOA	22	297
PFNA	11	21
PFDA	65	162
PFUnDA	22	26
PFDoDA	13	56
PFTrDA	11	18
PFTeDA	11	33
PFHxDA	22	23
PFOcDA	9834	9834
PFBS	10	19
PFPeS	10	10
PFHxS	26	80
PFHpS	10	10
PFOS	139	661
PFNS	21	21
PFDS	10	10
PFDoDS	21	21
PFECHS	10	10
FBSA	15	29
MeFBSA	210	210
FHxSA	22	22
MeFHxSA	51	69
FOSA	11	11
FPrPA (3:3 FTCA)	52	52
FPePA (5:3 FTCA)	22	22
FHpPA (7:3 FTCA)	22	22
FHUEA (6:2 FTUCA)	22	22
FOUEA (8:2 FTUCA)	11	11
FDUEA (10:2 FTUCA)	11	19
4:2 FTSA	5	5
6:2 FTSA	5	23
8:2 FTSA	11	11

10:2 FTSA	212	212
PFHxPA	52	52
PFOPA	212	212
PFDPA	11	11
6:6 PFPiA	50	50
6:8 PFPiA	3945	3945
8:8 PFPiA	9598	9598
11ClPF3OUdS (8:2 Cl-PFESA)	10	10
9ClPF3ONS (6:2 Cl-PFESA)	10	10
FOSAA	273	273
MeFOSAA	50	50
EtFOSAA	204	204
SAmPAP	255	255
diSAmPAP	268	268
6:2 mPAP	587	766
8:2 mPAP	504	504
10:2 mPAP	547	547
6:2 diPAP	182	241
8:2 diPAP	70	100
6:2/8:2 diPAP	390	518
10:2 diPAP	988	988
ADONA	7	20
HFPO-DA	22	22

6. Results of PFAS Analysis

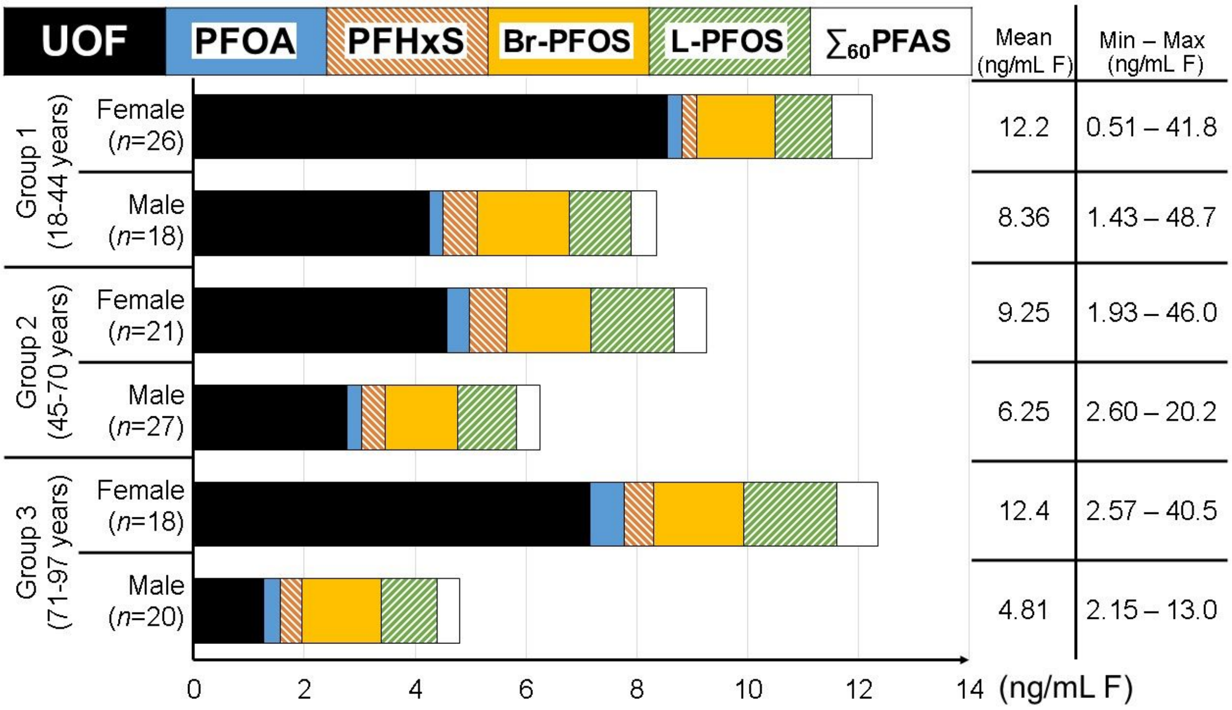

Figure S4. Organofluorine mass balance analysis as determined by EOF and target PFAS analysis in whole blood samples. The mass balance is made of unidentified organofluorine (UOF) and target PFAS. The values given are EOF concentrations (ng/mL F). $\sum_{60}$PFAS – all measured PFAS with the exception of PFOA, PFHxS and PFOS (linear + branched).

Table S4. Mean concentrations of PFCAs (ng/mL) and detection frequencies in whole blood samples.

	**Group 1 (18-44 years) Females (n = 33)**			**Group 1 (18-44 years) Males (*n* = 21)**			**Group 2 (45-70 years) Females (*n* = 24)**			**Group 2 (45-70 years) Males (*n* = 29)**			**Group 3 (71-97 years) Females (*n* = 20)**			**Group 3 (71-97 years) Males (*n* = 21)**		
	C (pg/mL)	n>LOQ	LOQ>n>LOD	C (pg/mL)	n>LOQ	LOQ>n>LOD	C (pg/mL)	n>LOQ	LOQ>n>LOD	C (pg/mL)	n>LOQ	LOQ>n>LOD	C (pg/mL)	n>LOQ	LOQ>n>LOD	C (pg/mL)	n>LOQ	LOQ>n>LOD
TFA		79%	0%		67%	0%		54%	4%		72%	0%		40%	0%		48%	0%
PFPrA		45%	0%		43%	5%		13%	4%		3%	14%		15%	15%		10%	10%
PFBA	<LOQ	0%	0%	<LOQ	0%	0%	<LOQ	0%	0%	<LOQ	0%	0%	17	10%	0%	17	10%	0%
PFPeA	<LOQ	0%	21%	<LOQ	0%	24%	12	4%	21%	<LOQ	0%	31%	2.6	5%	50%	4.5	5%	29%
PFHxA	<LOQ	0%	0%	<LOQ	0%	0%	<LOQ	0%	0%	<LOQ	0%	0%	<LOQ	0%	0%	<LOQ	0%	0%
PFHpA	<LOQ	0%	0%	<LOQ	0%	0%	<LOQ	0%	0%	<LOQ	0%	0%	3.6	5%	0%	2.7	5%	0%
PFOA	500	100%	27%	500	100%	19%	550	100%	29%	540	97%	31%	640	100%	5%	650	100%	14%
PFNA	200	100%	0%	230	100%	0%	290	100%	0%	290	93%	0%	340	100%	0%	310	100%	0%
PFDA	130	64%	3%	120	76%	0%	140	63%	13%	110	59%	0%	150	80%	5%	120	57%	10%
PFUnDA	100	52%	0%	90	52%	0%	88	54%	0%	43	24%	0%	88	50%	0%	47	43%	0%
PFDoDA	44	21%	0%	45	24%	0%	1.7	8%	0%	3.3	10%	0%	<LOQ	5%	0%	<LOQ	0%	0%
PFTrDA	6.6	21%	0%	10	24%	0%	4.2	13%	0%	1.8	7%	0%	<LOQ	5%	0%	1.4	14%	0%
PFTDA	28	15%	0%	4.8	5%	0%	<LOQ	0%	0%	<LOQ	3%	0%	<LOQ	0%	0%	<LOQ	0%	0%
PFHxDA	2.4	3%	0%	15	10%	0%	<LOQ	4%	0%	5.1	7%	0%	14	5%	0%	3.4	29%	0%
PFOcDA	<LOQ	0%	0%	<LOQ	0%	0%	<LOQ	0%	0%	<LOQ	0%	0%	<LOQ	0%	0%	<LOQ	0%	0%

Table S5. Mean concentrations of PFSAs and detection frequencies in whole blood samples.

	**Group 1 (18-44 years) Females (n = 33)**			**Group 1 (18-44 years) Males (*n* = 21)**			**Group 2 (45-70 years) Females (*n* = 24)**			**Group 2 (45-70 years) Males (*n* = 29)**			**Group 3 (71-97 years) Females (*n* = 20)**			**Group 3 (71-97 years) Males (*n* = 21)**		
	C (pg/mL)	n>LOQ	LOQ>n>LOD	C (pg/mL)	n>LOQ	LOQ>n>LOD	C (pg/mL)	n>LOQ	LOQ>n>LOD	C (pg/mL)	n>LOQ	LOQ>n>LOD	C (pg/mL)	n>LOQ	LOQ>n>LOD	C (pg/mL)	n>LOQ	LOQ>n>LOD
PFEtS	5.1	30%	0%	11	62%	0%	8.8	50%	0%	7.9	38%	0%	18	75%	0%	12	52%	0%
PFPrS	2.7	3%	0%	<LOQ	0%	0%	<LOQ	0%	0%	<LOQ	0%	0%	<LOQ	0%	0%	<LOQ	0%	0%
PFBS	4.8	18%	0%	4.1	38%	0%	2.3	29%	0%	2.7	17%	0%	5.1	35%	0%	4.5	33%	0%
PFPeS	9.8	61%	0%	167	57%	0%	9.1	33%	0%	18	59%	0%	8.1	50%	0%	2.9	24%	0%
PFHxS	320	100%	0%	880	100%	0%	600	100%	0%	730	100%	0%	440	100%	0%	680	100%	0%
PFHpS	250	97%	0%	280	100%	0%	200	96%	0%	300	100%	0%	230	100%	0%	370	100%	0%
Dimethyl-PFOS	41	24%	0%	41	38%	0%	210	29%	0%	140	31%	0%	120	35%	0%	140	33%	0%
3/4/5-*m*-PFOS	1200	100%	0%	1600	100%	0%	990	96%	0%	1500	100%	0%	900	100%	0%	2050	100%	0%
6/2-*m*-PFOS	350	100%	0%	430	100%	0%	350	96%	0%	420	100%	0%	350	100%	0%	600	100%	0%
1-*m*-PFOS	74	39%	0%	120	67%	0%	56	33%	0%	93	52%	0%	95	60%	0%	140	67%	0%
L-PFOS	1200	97%	0%	1500	100%	0%	1600	100%	0%	1800	100%	0%	1700	100%	0%	1900	100%	0%
PFNS	<LOQ	0%	0%	<LOQ	0%	0%	<LOQ	0%	0%	<LOQ	0%	0%	<LOQ	0%	0%	<LOQ	0%	0%
PFDS	<LOQ	0%	0%	<LOQ	0%	0%	<LOQ	0%	0%	<LOQ	0%	0%	<LOQ	0%	0%	<LOQ	0%	0%
PFDoDS	<LOQ	0%	6%	<LOQ	0%	10%	<LOQ	0%	4%	<LOQ	0%	17%	<LOQ	0%	0%	<LOQ	0%	10%

Table S6. Mean concentrations of PFSA precursors and detection frequencies in whole blood samples.

	**Group 1 (18-44 years) Females (n = 33)**			**Group 1 (18-44 years) Males (*n* = 21)**			**Group 2 (45-70 years) Females (*n* = 24)**			**Group 2 (45-70 years) Males (*n* = 29)**			**Group 3 (71-97 years) Females (*n* = 20)**			**Group 3 (71-97 years) Males (*n* = 21)**		
	C (pg/mL)	n>LOQ	LOQ>n>LOD	C (pg/mL)	n>LOQ	LOQ>n>LOD	C (pg/mL)	n>LOQ	LOQ>n>LOD	C (pg/mL)	n>LOQ	LOQ>n>LOD	C (pg/mL)	n>LOQ	LOQ>n>LOD	C (pg/mL)	n>LOQ	LOQ>n>LOD
FBSA	<LOQ	3%	0%	<LOQ	0%	0%	1.0	4%	0%	<LOQ	0%	0%	<LOQ	0%	0%	2.4	5%	0%
MeFBSA	<LOQ	0%	3%	<LOQ	0%	0%	2.1	0%	0%	<LOQ	0%	3%	<LOQ	0%	0%	<LOQ	0%	0%
FHxSA	3.8	3%	0%	<LOQ	0%	0%	6.4	13%	0%	3.0	14%	0%	1.6	5%	0%	<LOQ	0%	0%
MeFHxSA	<LOQ	0%	0%	<LOQ	0%	0%	<LOQ	0%	0%	<LOQ	0%	0%	<LOQ	0%	0%	<LOQ	0%	0%
FOSA	<LOQ	3%	0%	<LOQ	10%	0%	<LOQ	4%	0%	1.1	7%	0%	<LOQ	5%	0%	<LOQ	5%	0%
FOSAA	18	3%	0%	11	5%	0%	19	8%	0%	6.2	3%	0%	15	5%	0%	<LOQ	0%	0%
MeFOSAA	5.4	15%	0%	1.8	5%	0%	12	29%	0%	6.5	17%	0%	6.8	15%	0%	3.8	10%	0%
EtFOSAA	<LOQ	0%	0%	<LOQ	0%	0%	<LOQ	0%	0%	<LOQ	0%	0%	<LOQ	0%	0%	<LOQ	0%	0%
SAmPAP	<LOQ	0%	0%	<LOQ	0%	0%	<LOQ	0%	0%	<LOQ	0%	0%	<LOQ	0%	5%	<LOQ	0%	5%
diSAmPAP	<LOQ	0%	0%	<LOQ	0%	0%	<LOQ	0%	0%	<LOQ	0%	0%	<LOQ	0%	0%	<LOQ	0%	0%

Table S7. Mean concentrations of PFCA precursors and detection frequencies in whole blood samples.

	**Group 1 (18-44 years) Females (n = 33)**			**Group 1 (18-44 years) Males (*n* = 21)**			**Group 2 (45-70 years) Females (*n* = 24)**			**Group 2 (45-70 years) Males (*n* = 29)**			**Group 3 (71-97 years) Females (*n* = 20)**			**Group 3 (71-97 years) Males (*n* = 21)**		
	C (pg/mL)	n>LOQ	LOQ>n>LOD	C (pg/mL)	n>LOQ	LOQ>n>LOD	C (pg/mL)	n>LOQ	LOQ>n>LOD	C (pg/mL)	n>LOQ	LOQ>n>LOD	C (pg/mL)	n>LOQ	LOQ>n>LOD	C (pg/mL)	n>LOQ	LOQ>n>LOD
3:3 FTCA	<LOQ	0%	0%	<LOQ	0%	0%	<LOQ	0%	0%	<LOQ	0%	0%	<LOQ	0%	0%	<LOQ	0%	0%
5:3 FTCA	<LOQ	0%	0%	<LOQ	0%	0%	<LOQ	0%	0%	<LOQ	0%	0%	<LOQ	0%	0%	<LOQ	0%	0%
7:3 FTCA	<LOQ	0%	0%	<LOQ	0%	0%	<LOQ	0%	0%	<LOQ	0%	0%	<LOQ	0%	5%	<LOQ	0%	0%
6:2 FTUCA	<LOQ	0%	3%	<LOQ	0%	0%	<LOQ	0%	0%	<LOQ	0%	0%	<LOQ	0%	0%	<LOQ	0%	0%
8:2 FTUCA	<LOQ	3%	0%	<LOQ	0%	0%	<LOQ	0%	0%	<LOQ	0%	0%	<LOQ	0%	0%	<LOQ	0%	0%
10:2 FTUCA	<LOQ	3%	3%	<LOQ	0%	5%	<LOQ	4%	8%	5.3	3%	7%	<LOQ	5%	5%	<LOQ	0%	0%
4:2 FTSA	<LOQ	0%	0%	<LOQ	0%	0%	<LOQ	0%	0%	<LOQ	0%	0%	<LOQ	0%	0%	<LOQ	0%	0%
6:2 FTSA	2.1	15%	0%	2.2	10%	0%	<LOQ	0%	0%	<LOQ	7%	0%	<LOQ	0%	0%	<LOQ	0%	0%
8:2 FTSA	7.1	42%	0%	2.9	29%	0%	3.4	25%	0%	2.0	21%	0%	<LOQ	5%	0%	<LOQ	0%	0%
10:2 FTSA	<LOQ	0%	0%	<LOQ	0%	0%	<LOQ	0%	0%	<LOQ	0%	0%	<LOQ	0%	0%	<LOQ	0%	0%
6:2 mPAP	<LOQ	0%	0%	<LOQ	0%	0%	<LOQ	0%	0%	<LOQ	0%	0%	<LOQ	0%	0%	<LOQ	0%	0%
8:2 mPAP	<LOQ	0%	6%	<LOQ	0%	5%	<LOQ	0%	0%	<LOQ	0%	0%	<LOQ	0%	0%	<LOQ	0%	0%
10:2 mPAP	<LOQ	0%	0%	<LOQ	0%	0%	<LOQ	0%	0%	<LOQ	0%	0%	<LOQ	0%	0%	<LOQ	0%	0%
6:2 diPAP	<LOQ	0%	0%	<LOQ	0%	0%	<LOQ	0%	4%	<LOQ	0%	0%	<LOQ	0%	0%	<LOQ	0%	5%
8:2 diPAP	<LOQ	0%	0%	<LOQ	0%	0%	<LOQ	0%	0%	<LOQ	0%	0%	<LOQ	0%	0%	<LOQ	0%	0%
6:2/8:2 diPAP	<LOQ	0%	3%	<LOQ	0%	0%	<LOQ	0%	8%	<LOQ	0%	7%	<LOQ	0%	10%	<LOQ	0%	19%
10:2 diPAP	<LOQ	0%	0%	<LOQ	0%	0%	<LOQ	0%	0%	<LOQ	0%	0%	<LOQ	0%	0%	<LOQ	0%	0%

Table S8. Mean concentrations of PFPAs, PFPiAs, PFECHS, perfluoro ether acids and their detection frequencies in whole blood samples.

	**Group 1 (18-44 years) Females (n = 33)**			**Group 1 (18-44 years) Males (*n* = 21)**			**Group 2 (45-70 years) Females (*n* = 24)**			**Group 2 (45-70 years) Males (*n* = 29)**			**Group 3 (71-97 years) Females (*n* = 20)**			**Group 3 (71-97 years) Males (*n* = 21)**		
	C (pg/mL)	n>LOQ	LOQ>n>LOD	C (pg/mL)	n>LOQ	LOQ>n>LOD	C (pg/mL)	n>LOQ	LOQ>n>LOD	C (pg/mL)	n>LOQ	LOQ>n>LOD	C (pg/mL)	n>LOQ	LOQ>n>LOD	C (pg/mL)	n>LOQ	LOQ>n>LOD
PFHxPA	2.8	3%	0%	<LOQ	0%	0%	<LOQ	0%	0%	1.1	3%	0%	<LOQ	0%	0%	<LOQ	0%	0%
PFOPA	<LOQ	0%	0%	<LOQ	0%	0%	<LOQ	0%	0%	<LOQ	0%	0%	<LOQ	0%	0%	<LOQ	0%	0%
PFDPA	<LOQ	0%	0%	<LOQ	0%	0%	<LOQ	0%	0%	<LOQ	0%	0%	<LOQ	0%	0%	<LOQ	0%	0%
6:6 PFPiA	<LOQ	0%	0%	<LOQ	0%	0%	<LOQ	0%	0%	<LOQ	0%	0%	<LOQ	0%	0%	<LOQ	0%	0%
6:8 PFPiA	<LOQ	0%	0%	<LOQ	0%	0%	<LOQ	0%	0%	<LOQ	0%	0%	<LOQ	0%	0%	<LOQ	0%	0%
8:8 PFPiA	<LOQ	0%	0%	<LOQ	0%	0%	<LOQ	0%	0%	<LOQ	0%	0%	<LOQ	0%	0%	<LOQ	0%	0%
8:2 Cl-PFESA	<LOQ	0%	0%	<LOQ	0%	0%	<LOQ	0%	0%	<LOQ	0%	0%	<LOQ	0%	0%	<LOQ	0%	0%
6:2 Cl-PFESA	<LOQ	6%	0%	<LOQ	5%	0%	<LOQ	13%	0%	1.1	10%	0%	<LOQ	15%	0%	1.8	24%	0%
ADONA	1.8	6%	3%	4.6	19%	0%	2.2	8%	8%	1.5	7%	7%	1.1	5%	10%	<LOQ	5%	19%
HFPO-DA	<LOQ	0%	0%	<LOQ	0%	0%	<LOQ	0%	0%	<LOQ	0%	0%	<LOQ	0%	0%	<LOQ	0%	0%
PFECHS	10	58%	0%	18	81%	0%	20	88%	0%	24	83%	0%	20	90%	0%	20	95%	0%

7. Ratio of PFOS Isomers

Table S9. Ratio of branched and linear PFOS isomers in different demographic groups.

	C (ng/mL)					
	**Group 1 (18-44 years)**		**Group 2 (45-70 years)**		**Group 3 (71-97 years)**	
	**Females (*n* = 33)**	**Males (*n* = 21)**	**Females (*n* = 24)**	**Males (*n* = 29)**	**Females (*n* = 20)**	**Males (*n* = 21)**
Dimethyl-PFOS	0.04	0.29	0.21	0.14	0.12	0.14
3/4/5-*m*-PFOS	1.22	2.50	0.99	1.48	0.90	2.05
6/2-*m*-PFOS	0.35	1.45	0.35	0.42	0.35	0.60
1-*m*-PFOS	0.07	0.52	0.06	0.09	0.10	0.14
L-PFOS	1.19	2.33	1.57	1.80	1.73	1.89
Sum PFOS	2.87	7.09	3.18	3.93	3.2	4.82
%Br-PFOS	59%	67%	51%	54%	46%	61%
%L-PFOS	41%	33%	49%	46%	54%	39%

8. Results from NIST SRM1957

Table S10. Results from QC sample analysis.

Compound	This study (*n* = 19)		From CoA	
	Average (ng/mL)	CI[b] (k^c=2) (ng/mL)	Conc. (ng/mL)	CI[b] (k^c=2) (ng/mL)
PFHpA	0.256	0.085	0.305	0.051
PFOA	4.67	1.4	5.00	0.44
PFNA	0.843	0.26	0.878	0.077
PFDA	0.249	0.15	0.39	0.12
PFUnDA	0.109	0.089	0.172	0.036
PFHxS	4.07	1.9	4.00	0.83
∑PFOS[a]	18.0	3.9	21.1	1.3

[a]Sum of linear and branched PFOS isomers.
[b]Confidence interval.
[c]Coverage factor.

9. Bibliography

(1) Yeung, L. W. Y.; Robinson, S. J.; Koschorreck, J.; Mabury, S. A. Part I. A Temporal Study of PFCAs and Their Precursors in Human Plasma from Two German Cities 1982–2009. *Environ. Sci. Technol.* **2013**, *47* (8), 3865–3874. https://doi.org/10.1021/es303716k.
